# Differential Fairness Decisions and Brain Responses After Expressed Emotions of Others in Boys with Autism Spectrum Disorders

**DOI:** 10.1007/s10803-017-3159-4

**Published:** 2017-05-17

**Authors:** Eduard T. Klapwijk, Moji Aghajani, Gert-Jan Lelieveld, Natasja D. J. van Lang, Arne Popma, Nic J. A. van der Wee, Olivier F. Colins, Robert R. J. M. Vermeiren

**Affiliations:** 1 Department of Child and Adolescent Psychiatry, Curium - Leiden University Medical Center, Postbus 15, 2300 AA Leiden, The Netherlands; 2 Leiden Institute for Brain and Cognition (LIBC), Leiden, The Netherlands; 3 Department of Psychiatry, VU University Medical Center, Amsterdam, The Netherlands; 4 Institute of Psychology, Leiden University, Leiden, The Netherlands; 5 Department of Child and Adolescent Psychiatry, VU University Medical Center, Amsterdam, The Netherlands; 6 Institute of Criminal Law & Criminology, Faculty of Law, Leiden University, Leiden, The Netherlands; 7 Department of Psychiatry, Leiden University Medical Center, Leiden, The Netherlands

**Keywords:** Social decision-making, Autism spectrum disorders, Interpersonal effects of emotions, Dictator game, fMRI

## Abstract

Little is known about how emotions expressed by others influence social decisions and associated brain responses in autism spectrum disorders (ASD). We investigated the neural mechanisms underlying fairness decisions in response to explicitly expressed emotions of others in boys with ASD and typically developing (TD) boys. Participants with ASD adjusted their allocation behavior in response to the emotions but reacted less unfair than TD controls in response to happiness. We also found reduced brain responses in the precental gyrus in the ASD versus TD group when receiving happy versus angry reactions and autistic traits were positively associated with activity in the postcentral gyrus. These results provide indications for a role of precentral and postcentral gyrus in social-affective difficulties in ASD.

**Electronic supplementary material:**

The online version of this article (doi:10.1007/s10803-017-3159-4) contains supplementary material, which is available to authorized users.

## Introduction

Difficulties in reciprocal social interactions and communication are among the core features of autism spectrum disorders (ASD), along with a restricted repertoire of activities and interests (American Psychiatric Association 2013). These social deficits have been documented in numerous studies showing that individuals with ASD have impairments in the ability to represent other people’s mental states (i.e., mentalizing; Baron-Cohen et al. 1985; Kaland et al. 2008) and in processing emotions of others (Adolphs et al. 2001; Hobson 1986; Uljarevic and Hamilton 2013). Neuroimaging studies have also revealed differences between individuals with ASD compared to typically developing (TD) individuals in brain areas relevant for social-affective functioning (Di Martino et al. 2009; Fishman et al. 2014; Frith 2001; Pelphrey et al. 2011; Philip et al. 2012; White et al. 2014). These studies suggest that social deficits in ASD are associated with atypical activation in brain areas involved in mentalizing, such as hypoactivation in the medial prefrontal cortex (mPFC) and temporoparietal junction (TPJ) (e.g., Castelli et al. 2002; Wang et al. 2007; Watanabe et al. 2012), as well as in brain areas relevant for processing and resonating with others’ emotions such as hypoactivation in the inferior frontal gyrus and both under- and overactivation in the amygdala (e.g., Greimel et al. 2010; Klapwijk et al. 2016a; Monk et al. 2010; Pelphrey et al. 2007; Swartz et al. 2013).

In most of the neuroimaging studies on social processing in ASD, participants are merely required to observe others or to think about their mental states (e.g., Kana et al. 2015; Schulte-Ruther et al. 2011; Vander Wyk et al. 2014). Although these studies have greatly advanced the understanding of the neurocognitive mechanisms associated with social deficits in ASD, most do not take more interactive elements of social exchange into account. Studying such elements, however, is essential, as responding towards others involves different cognitive processes than merely observing others’ behavior (Schilbach et al. 2013). This is especially important because a discrepancy has been reported between potentially normative performance on explicit social tasks in ASD versus difficulties in applying social abilities during social interactions (Klin et al. 2003). For example, although adults with ASD do not spontaneously attribute mental states to others, they are able to understand mental states of others when they are explicitly encouraged to mentalize (Moran et al. 2011; Senju et al. 2009).

Paradigms inspired by behavioral economics are increasingly used to investigate social cognitive processes underlying social interactions in psychiatric populations (Hasler 2012; Sharp et al. 2012) including ASD (Chiu et al. 2008; Sally and Hill 2006; Yoshida et al. 2010). These paradigms not only offer simplicity and experimental control, but also have the advantage that they model interactive elements of social exchanges (King-Casas and Chiu 2012; Rilling and Sanfey 2011). Previous experiments using economic games suggest that people with ASD are indeed impaired in executing mentalizing abilities during interactive games. For example, adolescents with ASD show a different response in the middle cingulate cortex compared to controls when deciding to reciprocate investments in the trust game, suggesting problems with mentalizing during online social interaction (Chiu et al. 2008; Frith and Frith 2008). In a different strategic game, the stag hunt game, players can cooperate to hunt highly valued stags or act alone and hunt rabbits of lower value. Yoshida et al. (2010) used this game to estimate participants’ representations of the other player’s intentions for cooperation. They found that adults with ASD made less use of these representations than control participants when playing the game (Yoshida et al. 2010). Further evidence comes from a study in which children with ASD had to judge others’ morality and subsequently played a cooperative game both with the child they judged to be morally ‘nice’ and ‘bad’. This study showed that children with ASD (in contrast to TD children) did not distinguish between morally good and bad partners in the cooperative game but did correctly judge others’ morality in basic moral judgment stories (Li et al. 2014). These studies using economic games thus also suggest that individuals with ASD are able to make explicit inferences about others’ intentions but are less effective in using this information when making interactive decisions.

Although it has been suggested that individuals with ASD are impaired in processing emotions of others (Adolphs et al. 2001; Baron-Cohen et al. 1997; Harms et al. 2010), studies using economic games among individuals with ASD did not focus on the role of emotions in social interactions. However, many studies in healthy populations have shown that emotions expressed by others during interactions can influence subsequent behavior of the observer (van Kleef et al. 2010). For example, disappointed reactions of others might lead to fairer subsequent responses in observers than angry reactions of others (Lelieveld et al. 2012, 2013b), whereas during negotiations displays of happiness might signal satisfaction leading to lower offers (van Kleef et al. 2004). Currently, evidence suggests that individuals with ASD are less likely to integrate emotional contextual cues into their decision-making (De Martino et al. 2008). Yet little is known about how they make social decisions in response to emotions during social interaction. Therefore, in the current study we examined if emotions expressed by others influence fairness decisions and associated brain responses in boys with ASD compared with TD controls. While being scanned, participants were presented with written expressions of anger, disappointment and happiness by peers in response to an earlier decision about dividing tokens, after which they were given the opportunity to divide tokens again. A previous study using this paradigm found that TD adolescents reacted with more fair allocations after they read disappointed reactions compared with angry and happy reactions from their peers (Klapwijk et al. 2013). Neuroimaging studies that used this paradigm found that when TD participants received happy reactions they showed increased responses in the TPJ, a brain area that is important for mentalizing and attention (Klapwijk et al. 2016b; Lelieveld et al. 2013a).

Based on previous work showing that individuals with ASD made less use of social information when making decisions (Izuma et al. 2011; Li et al. 2014; Yoshida et al. 2010), we expected that they would be less likely to integrate emotional contextual information into their decision-making processes. This would be reflected in less differences in fairness decisions between the three emotions in the ASD versus TD group. Predictions for neuroimaging results were based on previous studies in ASD that revealed altered activation compared to controls in brain regions involved in social cognition. Whereas most previous studies used facial emotions, the current study used written emotions, and we therefore expected to find differences in frontotemporal brain regions involved both in social cognition and language processing. For example, reduced activation in the inferior frontal gyrus has been reported in ASD when presenting emotional faces (e.g., Baron-Cohen et al. 1999; Greimel et al. 2010; Holt et al. 2014) and altered activation in ASD in this region during mentalizing and social cognition has been identified in two meta-analyses (Di Martino et al. 2009; Philip et al. 2012). Furthermore, prior studies that used the same paradigm as in the current study showed that the TPJ is sensitive to happy reactions in TD controls (Klapwijk et al. 2016b; Lelieveld et al. 2013a). Given reports of reduced TPJ activation in social tasks in ASD (Castelli et al. 2002; Lombardo et al. 2011), we also expected group differences here.

## Method

### Participants

Male adolescents with ASD were recruited from specialized child psychiatric centers providing both inpatient and outpatient care for persons with ASD; TD control adolescents were recruited through local advertisement. All participants were aged 15–19 years (see Table 1 for participant characteristics). Exclusion criteria were (central) neurological abnormalities, a history of epilepsy or seizures, head trauma, left-handedness, and IQ less than 75. Intelligence was estimated using the Wechsler Adult Intelligence Scale—third edition (WAIS-III) or Wechsler Intelligence Scale for Children—third edition (WISC-III) subscales Vocabulary and Block Design.

**Table 1:** Group characteristics

	Autism spectrum disorders (ASD) (N = 19)	Typically developing (TD) (N = 19)	*p* value
Age, years (*SD*)	17.1 (1.2)	16.7 (1.2)	.38
IQ, *M* (*SD*)	107.7 (11.2)	102.7 (6.2)	.10
Empathy scores^a^			
Cognitive empathy, *M* (*SD*)	34.4 (3.9)	37.3 (5.3)	.16
Affective empathy, *M* (*SD*)	34.7 (7.9)	38.1 (6.6)	.06
SRS-A autistic traits, *M* (*SD*)*	66.7 (20.0)	35.1 (14.7)	<.001
YSR DSM-oriented scales^b^			
Depressive problems, *M* (*SD*)	6.5 (5.1)	3.7 (4.9)	.09
Anxiety problems, *M* (*SD*)	3.0 (2.3)	1.7 (1.4)	.05

*Significantly different at *p* < 0.001

^a^Self-report of affective and cognitive empathy was measured using the Basic Empathy Scale (Jolliffe and Farrington 2006)

^b^YSR is reported for *N* = 18 TD, due to missing data for one TD participant

The ASD group consisted of 23 adolescent boys with a clinical ASD diagnosis of whom 21 completed both phases of the task (see Experimental Task section below). Data from two ASD participants were discarded due to excessive motion, leaving a final sample of 19 participants with ASD. Two of the 19 boys were diagnosed with autistic disorder, nine with Asperger’s syndrome, and eight with pervasive developmental disorder not otherwise specified (PDD-NOS) according to the DSM-IV-TR criteria. In addition, according to diagnostic information from their clinicians, two participants also met DSM-IV-TR criteria for ADHD, two for dysthymia, and one for major depression. The autism diagnostic observational schedule-generic (ADOS-G; Lord et al. 2000) and autism diagnostic interview-revised (ADI-R; Lord et al. 1994) were administered besides clinical judgment. Seventeen participants met the criteria for autism or ASD on the Social Interaction and Communication domains of the ADOS-G, and two scored above the cut-off point only in one of these domains. However, these two participants fulfilled the ADI-R criteria for autism. We were able to administer the ADI-R for 17 participants and all 17 fulfilled the autism criteria on the ADI-R Social Interaction and Communications domains. Review of the medical charts of the other two indicated that autistic features were already present from an early age. Nine participants with ASD took medication at the time of testing (N = 1 atypical antipsychotics, N = 2 psychostimulants, N = 3 selective serotonin re-uptake inhibitors, N = 3 multiple medications). The social responsiveness scale self-report version (SRS-A) (Constantino and Gruber 2002; Constantino and Todd 2005) was used as a quantitative measure of autistic traits.

Thirty-seven TD control boys participated of whom 34 completed both phases of the task (see Experimental Task section below). Data from one TD participant was discarded due to excessive motion and another 14 for group-wise matching for age and IQ, leaving a final sample of 19 TD participants. All TD participants were screened using the SRS-A in order to exclude participants with heightened autistic traits (i.e., SRS-A *T*-score > 60). The youth self report (YSR; Achenbach 1991) was used to assess general psychopathology; data for one participant were missing but none of the other TD boys scored in the clinical range on the YSR externalizing or internalizing scales.

### Experimental Task

We examined participants’ fairness choices in the Dictator Game (Kahneman et al. 1986) after receiving emotional reactions from others, using a procedure previously used in studies with adults and (conduct disordered) adolescents (Klapwijk et al. 2013, 2016b; Lelieveld et al. 2013a). Participants first took part in a preliminary study 1 week before scanning (*first phase of the experiment*), where they read a scenario after which they were instructed to divide ten tokens between themselves and another person. They could choose a 6–4 distribution in favor of themselves, an equal distribution (5–5), or a distribution in favor of the other (4–6). This negotiation scenario was intended to assure that most participants chose the 6–4 option in this phase of the study. Only participants that chose a 6–4 distribution took part in the second phase of the experiment during scanning (21 out of 23 ASD boys and 34 out of 37 TD boys chose a 6–4 distribution). Hereby we assured the credibility of the second phase in which angry, disappointed, or happy emotional reactions would be directed at the 6–4 offer chosen in the first phase. Using these three emotions allows for comparisons of the effects of negative and positive communicated emotions and the effects of different types of negative emotions (Lelieveld et al. 2013a; van Kleef et al. 2010). Additionally, although it is not uncommon to find angry and disappointed reactions in response to a 6–4 distribution because of the relative unfairness of this distribution, happy reactions should be considered acceptable since offers of around 40% of the total are mostly accepted in economic games (Falk and Fischbacher 2006).

**Fig. 1:**
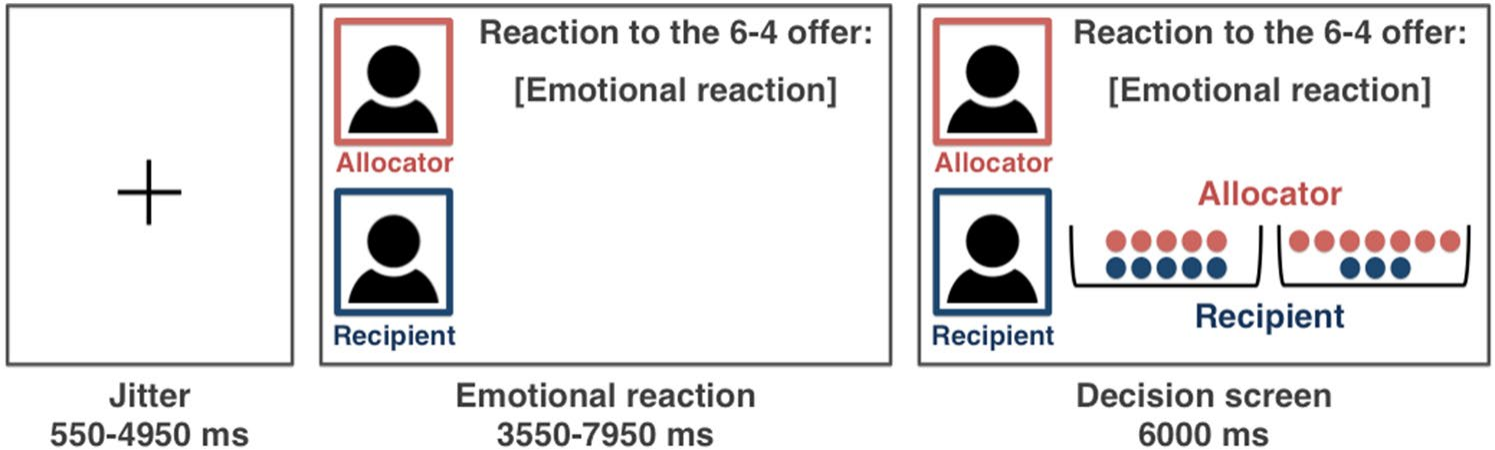
Visual display and timing (in milliseconds; ms) of the task in the scanner. The emotional reaction of the recipient (here “emotional reaction”) was displayed after a jittered fixation cross. Subsequently, the screen displayed two offers each containing red and blue tokens, which indicated the share for the allocator and the recipient, respectively (here 5–5 vs. 7–3). The name of the allocator was displayed in* red* (here “allocator”) and the name of the recipient in* blue* (here “recipient”). If participants did not respond within 6000 ms, a screen displaying ‘Too late!’ was presented. After the response, the decision screen remained on the screen until 6000 ms after the onset of the decision screen. (Color figure online)

In the *second phase of the experiment*, the boys were told that their unfair offer (the 6–4 distribution chosen in the first phase) was presented to 60 peers who were given the opportunity to write out their reaction upon receiving the offer. In reality, the reactions were preprogrammed and we left at least one week between the first and second phase to increase the credibility that researchers actually collected reactions from others. During scanning, participants were paired with a different player on each trial, whose first name was provided and whose reaction to the 6–4 distribution was angry, disappointed, or happy. Participants read the reactions of their peers and subsequently played a version of the Dictator Game with the peer who provided the reaction (see Fig. 1). In this Dictator Game the participants were the allocator and had to divide ten tokens. They could choose between different fair and unfair distributions while the recipient had to accept any distribution they would make. Each trial started with a jittered fixation (min = 0.55 s, max = 4.95 s, M = 1.54 s), after which the participants were presented with the emotional reaction for a period of 3 s plus a jittered interval (min = 0.55 s, max = 4.95 s, M = 1.54 s) and subsequently had 6 s to make a decision between two distributions. The 60 trials were presented in pseudo-random order divided over three blocks of 4 min each. Before the task started, participants learned that at the end of the experiment the computer would randomly select ten trials to determine their total earnings, which would be added to the standard compensation for their participation. At the end of the session, participant’s pay-off was presented, which varied between 2.5 and 6 euros. Afterwards, participants completed a questionnaire in which they were probed for suspicion. None of the participants expressed doubt about the set-up of the task.

### fMRI Data Acquisition

Imaging was carried out at the Leiden University Medical Center on a 3 T Philips Achieva MRI scanner. Prior to scanning, participants were familiarized with the scanner environment using a mock scanner. For fMRI, T2* weighted gradient echo, echo planar images (EPI) sensitive to BOLD contrast were obtained with the following acquisition parameters: repetition time (TR) = 2.2 s, echo time (TE) = 30 ms, flip angle = 80°, 38 axial slices, field of view (FOV) = 220 × 220 mm, 2.75 mm isotropic voxels, 0.25 mm slice gap. Data from participants with excess motion defined by relative mean displacement > 0.5 mm were excluded from further analysis (ASD N = 2; TD N = 1). A high-resolution anatomical image (T_1_-weighted ultra-fast gradient-echo acquisition; TR = 9.75 ms, TE = 4.59 ms, flip angle = 8°, 140 axial slices, FOV = 224 × 224 mm, in-plane resolution 0.875 × 0.875 mm, slice thickness = 1.2 mm) was acquired for registration purposes. All anatomical scans were reviewed by a radiologist; no anomalies were found.

### fMRI Data Analysis

FMRI data analysis was conducted using FEAT (fMRI expert analysis tool) version 6.00, part of FSL (http://www.fmrib.ox.ac.uk/fsl). The following prestatistics processing was applied: motion correction using MCFLIRT, non-brain removal using BET, spatial smoothing using a Gaussian kernel of FWHM 5 mm, grand-mean intensity normalization of the entire 4D dataset by a single multiplicative factor, and high-pass temporal filtering (Gaussian-weighted least-squares straight line fitting, with sigma = 50.0 s). Functional scans were registered to the T1-weighted anatomical images, and subsequently to the 2 mm MNI-152 standard space template. Time-series statistical analysis was performed using FILM with local autocorrelation correction. To investigate the effects of the communicated emotions, we modeled the onset of the presentation of the three different emotional reactions (i.e., anger, disappointment, happiness) as an event with zero duration convolved with a gamma hemodynamic response function. To account for residual movement artifacts, the six realignment parameters (three for translation in mm and three for rotation in degrees) were included in the model as covariates of no interest. Note that in the final sample used in the present study there were no significant differences in the six realignment parameters (all *p* > 0.05) between the ASD and TD groups. At first-level for each run for each participant, primary contrasts of interest were generated. Positive versus negative emotions were contrasted [happiness > [(anger and disappointment)] as well as happiness against the separate negative emotions (happiness > anger; happiness > disappointment) and the negative emotions against each other (anger > disappointment). A second-level, fixed-effects analysis combined data across the three runs for each participant. Individual participant data were then entered into a third-level group analysis using a mixed-effects design (FLAME) whole-brain analysis. The general linear model included the two groups (ASD and TD) and to account for possible age effects, we included age (mean-centered) as covariate of no interest. In addition, in the ASD group we analyzed the effects of autistic traits on brain responses during the different contrasts by using SRS scores as regressors of interest, adding age (mean-centered) as covariate of no interest. Resulting statistical maps were corrected for multiple comparisons using cluster-based correction (*p* < .0.05, initial cluster-forming threshold *Z* > 2.3). We used Featquery and SPSS version 22 (IBM Corp., Armonk, NY, USA) to conduct region of interest (ROI) analyses to correlate task behavior and ASD symptom scores with patterns of activity from regions that were identified in the whole-brain analyses. Functional ROIs from these regions were generated by masking the activation maps of the contrasts of interest with binarized anatomical ROIs using the Harvard-Oxford structural atlases distributed with FSL. Finally, we explored whether additional clinical factors, such as medication exposure or comorbidity, might have influenced the results. Extracted *z* values from the ROIs identified in the whole-brain analyses were entered into SPSS to compare only those participants with ASD without a comorbid disorder, those not using medication, or both to TD controls. Additionally, we compared ASD participants with a comorbid disorder to those without and ASD participants who were on medication to those who were not. Given the high rates of anxiety reported in ASD (White et al. 2009) and the possible impact of anxiety on social decision making (Luo et al. 2014; Wu et al. 2013), we also repeated the fMRI analyses with YSR DSM-oriented Anxiety problems as a covariate of no interest to account for possible effects of anxiety. Mean group substitution was used to replace missing YSR data for one TD participant.

## Results

### Behavioral Results

**Fig. 2:**
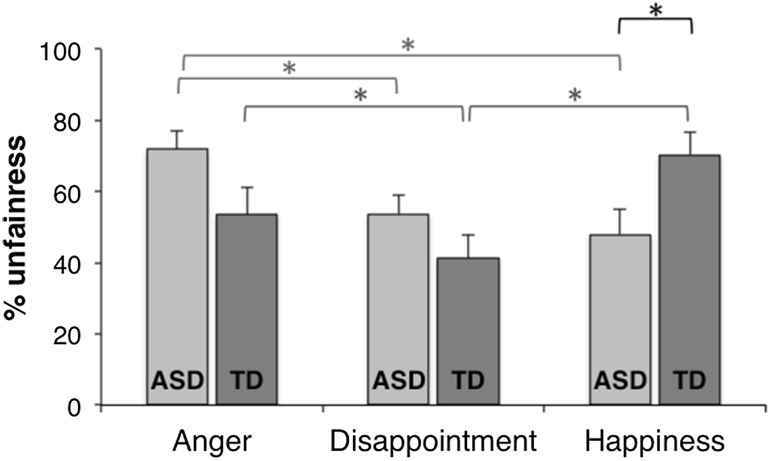
Percentage of unfair offers after communication of anger, disappointment, and happiness, separate for ASD and TD groups. *Asterisks* indicate significant differences within groups (*green* ASD; *blue* TD) and between groups (in *black*). (Color figure online)

Fairness decisions after the three different emotions were compared between the groups with a 2 × 3 mixed ANOVA (group × emotion). We found a main effect of emotion, *F* (1, 37) = 4.48, *p* = .015, caused by a higher percentage of unfair offers in response to angry (*M* = 62.7%; *SD* = 29.9, *p* < .001) and happy (*M* = 59.1%; *SD* = 31.0, *p* < .05) compared to disappointed reactions (*M* = 47.5%; *SD* = 26.0). There was no main effect of group, *F* (1, 37) = 0.18, *p* = .68, showing that the groups did not differ on fairness levels across the emotions combined. We found a significant interaction effect, *F* (1, 37) = 8.52, *p* < .001, showing group differences in the reactions after the different emotional expressions (see Fig. 2). Post hoc tests revealed that within the ASD group, participants more often chose the unfair than the fair option when dealing with angry peers (71.8%, SD = 22.7) than when dealing with disappointed (53.7%, SD = 23.1, *p* = .001) and happy (47.9%, SD = 31.1, *p* < .05) peers. No differences in fairness decisions after disappointment and happiness were found in the ASD group. Next, within the TD group, we found that participants more often chose the unfair than the fair option when dealing with angry (53.6%, SD = 33.8, *p* < .01) and happy (70.3%, SD = 27.2, *p* < .005) peers than when dealing with disappointed peers (41.3%, SD = 27.8). No differences in fairness decisions after anger and happiness were found in the TD group. Finally, between-group comparisons showed that the ASD (versus TD) group made significantly less unfair offers after happy reactions (*p* < .05), and marginally significantly more unfair offers after angry reactions (*p* = .058). No significant group difference was found in unfairness after disappointed reactions (*p* = .14).

### fMRI Results

**Fig. 3:**
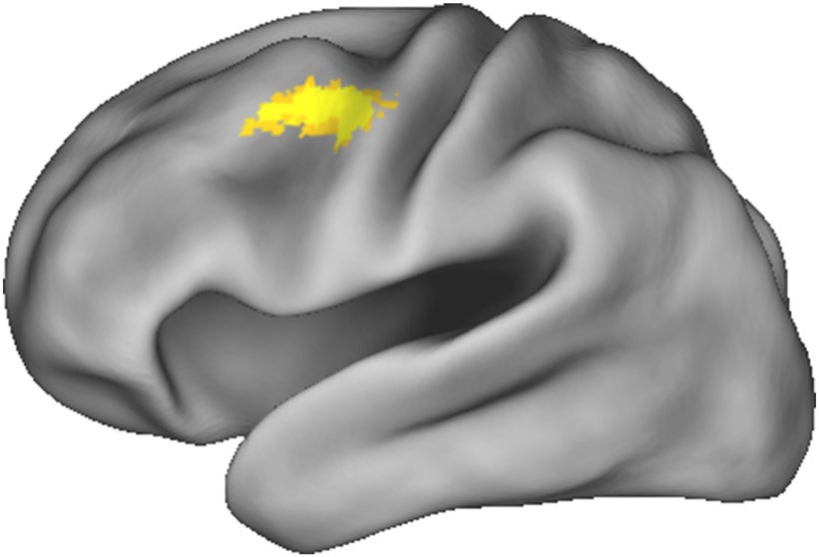
Group differences in the left precentral gyrus for the happiness > anger contrast cluster-thresholded at *z* > 2.3, *p* < .05

The first set of whole-brain analyses aimed to identify regions that differed between the ASD and TD groups when receiving positive relative to negative emotional reactions in general [i.e., happiness > (anger and disappointment) contrast]. No group differences were found whilst using this contrast. When analyzing the contrasts that compared happiness to a specific negative emotion (i.e., happiness > anger, and the happiness > disappointment) we found that the ASD (vs. TD) group showed less activation in the left and right precentral gyrus and right middle frontal gyrus in the happiness > anger contrast (see Table 2 and Fig. 3). Finally, when comparing the two negative emotions with each other, we found no significant group differences between the ASD and TD groups when analyzing the anger > disappointment and disappointment > anger contrasts.

**Table 2:** MNI coordinates, *z* values and cluster sizes for brain regions revealed by the whole brain pairwise comparisons of the TD control > ASD groups, *z* > 2.3, *p* < .05 cluster-corrected

Anatomical region	Max *z*	MNI peak coords			Size in voxels
		x	y	z	
TD > ASD					
Happiness > anger					
L precentral gyrus	3.94	−44	2	46	396
R precentral gyrus	3.59	56	2	42	376
R middle frontal gyrus	3.36	54	32	26	(Part of above)
Autistic traits (ASD group only)					
Happiness > [anger and disappointment]					
L postcentral gyrus	3.71	−48	−32	52	1386
Happiness > disappointment					
L postcentral gyrus	3.71	−48	−32	56	1056
Happiness > anger					
L supramarginal gyrus	3.45	−46	−44	56	398
L postcentral gyrus	3.45	−46	−28	50	(Part of above)

Activation clusters were labeled using the Harvard-Oxford structural atlases

**Fig. 4:**
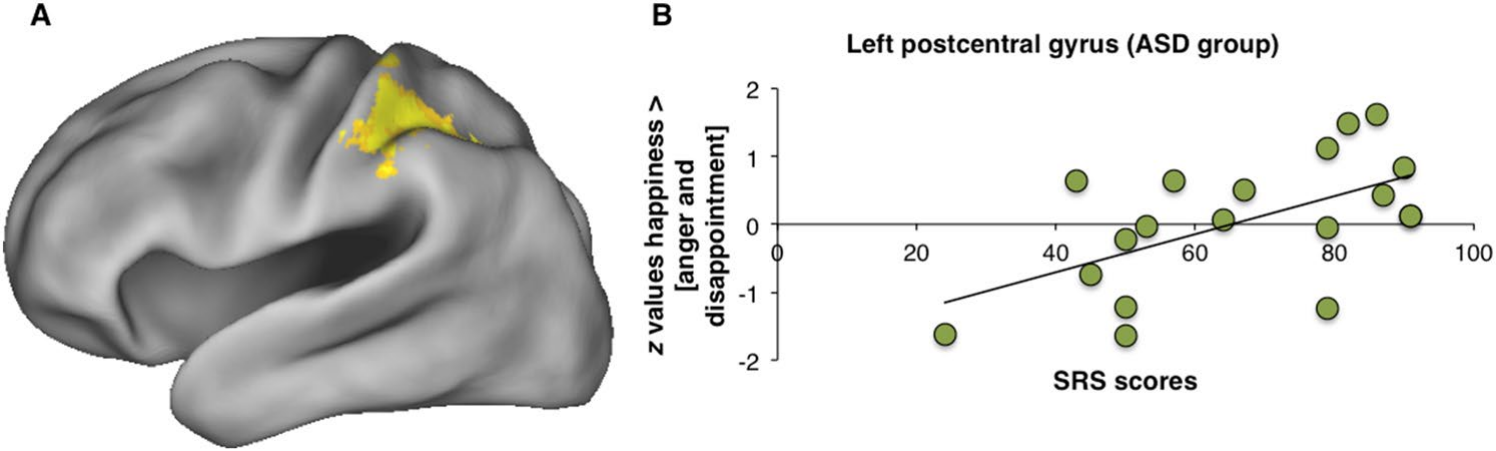
**a** Higher autistic traits in the ASD group were related to higher activity in the left postcentral gyrus in the happiness > [anger and disappointment] contrast cluster-thresholded at *z* > 2.3, *p* < .05 with **b** graph showing mean *z* values in the postcentral gyrus as a function of SRS scores in the ASD group

We also analyzed the effects of autistic traits as measured by the SRS-A on brain responses during the different contrasts in the ASD group separately. These analyses revealed that higher activity in the left postcentral gyrus and supramarginal gyrus in the happiness > [anger and disappointment] contrast was related to higher autistic traits in the ASD group (see Fig. 4). This relation was also found between autistic traits and activity in these regions in the separate happiness > anger and happiness > disappointment contrasts. No other brain regions showed an association between autistic traits and activity in any of the contrasts. Additionally, control analyses showed no relation between autistic traits and brain activation in the TD group, suggesting the relation between autistic traits and brain activation is specific for the ASD group. We also repeated the fMRI analyses with the lowest scoring participant removed, which also showed a relation between autistic traits and activity in the left postcentral gyrus (see Supplementary materials).

### Relationships Between Fairness Decisions and Brain Activation

Next, we conducted exploratory analyses to investigate the relation between fairness decisions and brain activity in regions identified in our whole-brain analysis. We investigated the relation between the percentage of unfair offers in response to happy reactions and activity in the right precentral gyrus for the happiness > anger contrast. This analysis revealed a significant negative correlation between the percentage unfair offers and left precental gyrus activity for the TD control group (*r* = −0.56, *p* < 0.5), but not for the ASD group (*r* = 0.08, *p* = 0.75). However, Fisher z-values were calculated which indicated that the difference between these correlations was not significant (*z* = 1.56, *p* = 0.58).

### Effects of Comorbidity and Medication

Post-hoc analyses revealed that all group differences remained significant when excluding ASD participants with comorbid disorders or those using medication (all *p*s < .01). In addition, no significant group differences were found between ASD participants with comorbid disorders and those without (all *p*s > .2) or between ASD participants using medication or not (all *p*s > .6). The analyses with the YSR DSM-oriented Anxiety problems as a covariate did not considerably alter results. Only minor changes in size and peak coordinates of the clusters revealed in the main analysis were observed (see Supplemental Table S1).

## Discussion

This is the first study focusing on the effects of emotions on fairness decisions and brain responses in ASD. Behavioral analyses showed that ASD participants were more unfair when dealing with angry compared to disappointed and happy peers, whereas TD participants more often were unfair when dealing with angry but also with happy peers compared to those that communicated disappointment. These group differences were mainly driven by differences in reactions to happy peers, as the TD group chose significantly more unfair offers after happy reactions than the ASD group. The imaging results showed reduced brain responses in the precental gyrus and middle frontal gyrus in the ASD versus TD group when receiving happy versus angry reactions. Additionally, more autistic traits in the ASD group were associated with more activity in the postcentral gyrus in the happiness versus anger and disappointment contrasts.

Although we hypothesized that the ASD group would be less likely to differentiate between the three emotions when making fairness decisions, this hypothesis was not supported as the behavioral results suggest that individuals with ASD did adjust their allocation behavior in response to the emotions of others. However, participants with ASD reacted less unfair than TD controls in response to happiness (and more unfair in response to anger compared to TD controls, although this difference failed to reach significance). The increase in unfairness in response to happiness of the TD participants is in line with findings from previous studies (Klapwijk et al. 2016b, 2013; van Kleef et al. 2004). When receiving a happy reaction after a previous unfair offer, one could infer that the other was already satisfied and would therefore be content with another unfair offer (Cacioppo and Gardner 1999; van Kleef et al. 2010). Possibly, our participants with ASD used different heuristics that require less such inferences about mental states since they did not choose to be more unfair in response to happiness compared to the TD participants. However, this interpretation could not be supported by altered activation in brain regions usually associated with mentalizing in the ASD group in the current study.

We did not find group differences in the specifically hypothesized brain regions that have been previously linked to atypical social-affective functioning in ASD such as the IFG and TPJ (Greimel et al. 2010; Lombardo et al. 2011). The absence of group differences in these areas might result from the specific task used in the current study, in which written emotions were presented and participants made fairness decisions subsequently. However, previous studies did report differences between ASD and TD controls in these regions in tasks using written stimuli (Lombardo et al. 2011) and the TPJ specifically has been implicated in previous studies using the same paradigm as in the current study (Klapwijk et al. 2016b; Lelieveld et al. 2013a). It might also be that individuals with ASD do not recruit these hypothesized social-affective brain regions differently from controls when making social decisions. The only other study that used fMRI to study social decisions in an economic game in ASD found group differences between individuals with ASD and controls in the middle cingulate gyrus (Chiu et al. 2008), and not in either IFG, mPFC, TPJ or amygdala. Given the sparse number of neuroimaging studies that employed economic games in ASD and the posited potential for understanding mental disorders using neuroeconomics (Hasler 2012; King-Casas and Chiu 2012; Kishida et al. 2010; Sharp et al. 2012), future studies are warranted to further test which brain regions are differentially recruited when making social decisions in ASD.

Interestingly, however, the reduced responses observed in the current study in the precentral gyrus and middle frontal gyrus, and also the postcentral gyrus activation related to autistic traits, align with results from recent meta-analyses of fMRI studies in ASD (Di Martino et al. 2009; Dickstein et al. 2013; Patriquin et al. 2016). Hypoactivation during social tasks in ASD versus controls was found in both the left and right precentral gyrus in the meta-analysis by Di Martino et al. (2009) and in the left precentral gyrus in the Patriquin et al. (2016) meta-analysis. Reduced responses in this area in ASD versus controls have been reported during imitation of emotional expressions and finger movements (Dapretto et al. 2006; Williams et al. 2006) and when observing fearful expressions (Deeley et al. 2007). Although the precentral gyrus is considered to be part of motor-related cortex, activity in this area has previously been associated with social-emotional functioning. Precentral gyrus activity has been found to increase when receiving empathic responses from others (Seehausen et al. 2014, 2016) and activity in this area is also related to self-reported affective empathy in social versus nonsocial emotional scenes (Hooker et al. 2010). Furthermore, atypical functional connectivity within the precentral gyrus has been associated not only with impaired motor skills but also with social deficits in ASD (Nebel et al. 2014). In the current study, reduced activation in the precental gyrus was found in the ASD versus TD group specifically when contrasting happy versus angry reactions. This might suggest that the ASD participants process the happy emotional information differently than the TD controls in this area and therefore also responded less unfair in response to happiness than the TD group. However, future studies are needed to further clarify the role of the precentral gyrus in social-emotional functioning. For example, the current paradigm does not allow inferring whether the different response to happiness in the ASD group is the result of less responsiveness to happy emotions in general or to a different cognitive appraisal of happiness that leads to increased fairness and decreased precentral gyrus activation. Experiments in which the emotional intensity of happiness is varied could resolve whether responsiveness to happiness is related to precentral gyrus activation or not. The current findings as well as the precentral gyrus hypoactivation in ASD during social tasks in two meta-analyses (Di Martino et al. 2009; Patriquin et al. 2016) might point to a relation between precentral gyrus dysfunctions and social deficits in ASD.

The current results additionally showed a positive association between autistic traits and activity in the postcentral gyrus in the ASD group in the happiness versus anger and disappointment contrasts. The postcentral gyrus is a somatosensory region that is also not usually discussed in the context of ASD social deficits, although it has consistently been revealed as a hyperactivated region in ASD meta-analyses of social tasks (Di Martino et al. 2009; Dickstein et al. 2013; Patriquin et al. 2016) and it has also been reported as a region being structurally altered in ASD (Hyde et al. 2010). Previous studies in healthy populations have reported the involvement of primary somatosensory cortex in affective touch (Gazzola et al. 2012), in processing facial and vocal emotions (Adolphs et al. 2000; Heberlein and Atkinson 2009) and in affective language use (Saxbe et al. 2013). The relation between autistic traits and postcentral gyrus activation in response to happy versus angry and disappointed emotions in the current paradigm might suggest a specific relation between somatosensory processing of positive emotions and ASD symptoms.

Several limitations to this study should be noted. First, although our sample size (N = 19 per group) is comparable with other task-related fMRI studies in ASD, this sample size is relatively small and may have limited the power to detect group differences in brain regions usually linked to social cognition and emotion processing. Second, since our sample contained adolescent boys only, we do not know whether our results are generalizable to girls and to children and adults with ASD. Third, the task design employed in the current study contained written preset emotions only. Future studies could further increase the amount of interaction by studying face-to-face interactions, for example by using virtual reality. Finally, it remains unclear why differences in ASD versus controls were found in the precentral gyrus, whilst a correlation with autistic traits was found in the postcentral gyrus but not in the precentral gyrus. It can be speculated that the relatively small sample size has limited the power to find a correlation between precentral gyrus activation and autistic traits. It is also possible that a correlation between autistic traits and brain activity within the ASD group does not necessarily imply group differences in the same region between the ASD and TD groups.

In conclusion, the current study provides an initial step in examining how explicit emotional feedback influences interactive decisions and associated brain responses in ASD. The results suggest that individuals with ASD do employ explicitly expressed emotional information when making social decisions, although responses towards happiness seemed atypical and were fairer than controls. The neuroimaging results might point to a possible role of precentral and postcentral gyrus in social-affective difficulties in ASD, although more research is needed to specify the neurocognitive mechanisms that are associated with these brain regions during social cognition. Future research in which the role of others’ expressed emotions is further investigated could help to refine models for social interactions in ASD.

## Electronic supplementary material

Below is the link to the electronic supplementary material.

Supplementary material 1 (DOCX 74 KB)
