# Supplementary material for: Differential Fairness Decisions and Brain Responses After Expressed Emotions of Others in Boys with Autism Spectrum Disorders
Source: J Autism Dev Disord. 2017 May 17;47(8):2390–400. doi: 10.1007/s10803-017-3159-4 (PMC5509841; doi:10.1007/s10803-017-3159-4)
Supplement: Supplementary file 1 — Supplementary material 1 (DOCX 74 KB) [file 10803_2017_3159_MOESM1_ESM.docx]

**Supplementary materials**

**Table S1.** MNI coordinates, *z* values and cluster sizes for brain regions revealed by the whole brain pairwise comparisons of the TD control > ASD groups including YSR DSM oriented Anxiety problems as a covariate, *z* > 2.3, *p* < .05 cluster-corrected. Activation clusters were labeled using the Harvard-Oxford structural atlases.

| **Anatomical region** | **Max *z*** | **MNI peak coords** | | | **Size in voxels** |
| --- | --- | --- | --- | --- | --- |
|  |  | **x** | **y** | **z** |  |
| **TD > ASD** | | | | |  |
| *happiness > anger* | | | | |  |
| L precentral gyrus | 3.97 | -52 | 4 | 46 | 388 |
| R middle frontal gyrus | 3.62 | 52 | 28 | 34 | 425 |
| R precentral gyrus | 3.36 | 56 | 2 | 42 | (part of above) |
| **Autistic traits (ASD group only)** | | | | | |
| *happiness > [anger and disappointment]* | | | | | |
| L postcentral gyrus | 3.67 | -48 | -32 | 52 | 1207 |
| *happiness > disappointment* | | | | | |
| L postcentral gyrus | 3.68 | -48 | -32 | 56 | 918 |

**Table S2.** MNI coordinates, *z* values and cluster sizes for brain regions revealed by the whole brain analysis with autistic traits as covariate with outlier removed (*N* = 18), *z* > 2.3, *p* < .05 cluster-corrected. Activation clusters were labeled using the Harvard-Oxford structural atlases.

| **Anatomical region** | | | | **Max *z*** | | **MNI peak coords** | | | | | | **Size in voxels** | | |
| --- | --- | --- | --- | --- | --- | --- | --- | --- | --- | --- | --- | --- | --- | --- |
|  | | | |  | | **x** | | **y** | | **z** | |  | | |
| **Autistic traits (ASD group only, outlier removed, *N* = 18)** | | | | | | | | | | | |  | | |
| *happiness > [anger and disappointment]* | | | | | | | | | | |  | | |  |
| L middle frontal gyrus | | 3.83 | | -28 | | 36 | | 44 | | 679 | | |  |  |
| L postcentral gyrus | | 3.52 | | -48 | | -32 | | 52 | | 929 | | |  |  |
| Paracingulate gyrus | | 3.39 | | 2 | | 28 | | 34 | | 539 | | |  |  |
| L precentral gyrus | | 3.26 | | -48 | | 8 | | 28 | | 318 | | |  |  |
| *happiness > anger* | | | | | | | | | | | |  | | |
| Paracingulate gyrus | | 3.88 | | 4 | | 26 | | 34 | 628 | |  |  |  |  |
| L supramarginal gyrus | | 3.32 | | -46 | | -44 | | 56 | | 454 | | |  |  |
| L postcentral gyrus | | 3.10 | | -46 | | -28 | | 50 | | (part of above) | | |  |  |
